# The Frequency of Hypoglycemia and Its Symptoms in Preterm Neonates in the First 24 Hours

**DOI:** 10.7759/cureus.62356

**Published:** 2024-06-14

**Authors:** Jehanzeb Khan, Qazi Muhammad Safwan, Hanadi Shad, Adnan Shah, Ayesha Johar, Palwasha Rasool, Yaseen Khan, Syed Hassnain Shah, Adnan Khan, Rao E Hassan

**Affiliations:** 1 Pediatric Medicine, Hayatabad Medical Complex, Peshawar, PAK; 2 Internal Medicine, Hayatabad Medical Complex, Peshawar, PAK; 3 Internal Medicine, Lady Reading Hospital, Peshawar, PAK; 4 Pediatrics, Lady Reading Hospital, Peshawar, PAK; 5 Pediatrics, Khalifa Gul Nawaz Teaching Hospital, Bannu, PAK; 6 Orthopedics and Trauma, Pakistan Institute of Medical Sciences, Islamabad, PAK; 7 Orthopedics and Trauma, Khyber Teaching Hospital Medical Teaching Institute (MTI), Peshawar, PAK

**Keywords:** low birth weight, hypoglycemia workup, cross-sectional, neonatal hypoglycemia, glucose metabolism, preterm neonate

## Abstract

Introduction

Hypoglycemia is a critical concern in neonatal care, particularly among preterm infants. This study aims to investigate the frequency of hypoglycemia within the first 24 hours of life in preterm neonates, considering factors such as gestational age (GA), birth weight, and gender.

Materials and methods

A cross-sectional study was conducted from February to August 2021. The sample comprised 186 preterm infants selected through consecutive sampling. Data collection involved demographic information, glucose level monitoring, and symptom assessment.

Results

Of the 186 preterm neonates, 31.7% (n=59) experienced hypoglycemia within the first 24 hours, with feeding refusal being the predominant symptom. There was a significant difference in hypoglycemia occurrence between infants born before and after 32 weeks of gestation (p<0.05). Males were slightly more affected than females, although not statistically significant. Infants weighing less than 2 kg showed a higher susceptibility to hypoglycemia.

Conclusion

The early detection and management of hypoglycemia are crucial in preterm neonatal care. Close monitoring, especially in the initial four hours, is essential to prevent complications. Larger studies are warranted to confirm these findings and improve understanding and management strategies for hypoglycemia in preterm neonates, particularly within the first 24 hours of life.

## Introduction

Hypoglycemia is the decreased level of blood glucose that is insufficient for the body's needs [1]. The American Academy of Pediatrics (AAP) and the Pediatric Endocrine Society (PES) have developed guidelines for safe blood sugar levels in newborns. These guidelines acknowledge that an infant's blood sugar can drop as low as 30 mg/dL within the first two hours after birth but should then rise to at least 45 mg/dL before stabilizing around 12-24 hours [2].

Early in gestation, fetal and maternal glucose levels correlate significantly. However, by the third trimester, fetal glucose utilization rises, leading maternal levels to exceed fetal levels. Preterm infants usually reflect their mothers' serum glucose concentrations. In severely growth-restricted fetuses, the gradient widens to aid diffusion, influenced by clinical severity [3,4]. While enzymes for gluconeogenesis are present from early pregnancy, the fetus generates its own glucose mainly during maternal starvation [5], suggesting that fetal blood glucose levels correlate with both gestational age (GA) and maternal glucose levels.

Neonatal hypoglycemia is essentially the inability to adapt to extrauterine physiology. It results from an imbalance between glucose supply and utilization due to immature regulatory mechanisms [6]. In a New Zealand study, approximately 50% of infants developed hypoglycemia, with 19% experiencing severe episodes and the same percentage having multiple episodes. On average, each episode lasted 1.4 hours, with the majority (81%) occurring within the first 24 hours [7]. A study in Pakistan found hypoglycemia in 112 out of 292 neonates (38.4%) [8].

Preterm infants undergo unique physiological adaptations during the transition from fetal to neonatal life. Despite lower catecholamine levels at birth in cesarean deliveries compared to vaginal births, some preterm infants paradoxically exhibit higher levels than term infants. These catecholamines play a vital role in maintaining blood pressure, promoting thermoregulation, and preventing hypoxia. Additionally, a cortisol surge accompanies catecholamine release in preterm infants, leading to increased gluconeogenesis, which contributes to elevated serum glucose levels. However, while preterm infants typically demonstrate higher cortisol levels than term infants, extremely premature or critically ill infants may not exhibit the same cortisol response, increasing their susceptibility to hypoglycemia [2]. Preterm newborns rely on their limited glycogen stores to regulate glucose levels after birth. Unlike full-term infants, who have accumulated substantial glycogen by term, preterm infants have smaller reserves, making them more vulnerable to hypoglycemia shortly after delivery. Moreover, preterm birth itself, often associated with increased prenatal stress, further compounds their susceptibility to hypoglycemia [9].

The objective of this study was to determine the frequency of hypoglycemia in preterm infants within the first 24 hours of life. As no prior research has examined this age group and timeframe together, this study will contribute significantly to the literature. The findings can inform the development of strategies for managing hypoglycemia during the critical first 24 hours of life, ultimately reducing morbidity in both the short and long term.

## Materials and methods

This cross-sectional study was conducted at the Department of Pediatrics Medicine of Hayatabad Medical Complex, Peshawar, from February 15, 2021, to August 15, 2021. The Ethical Committee of Hayatabad Medical Complex, Peshawar, issued approval 239/HEC/B&PC. The sample size, determined with a 95% confidence level, a 7% margin of error, and a 38.4% prevalence of hypoglycemia in preterm babies [8], comprised 186 infants selected through non-probability consecutive sampling. The inclusion criteria included neonates aged 24 hours or less, all preterm babies, and parental consent. The exclusion criteria encompassed term babies, those with ambiguous genitalia, infants over 24 hours old, and parental refusal of consent.

Neonatal hypoglycemia was defined as blood glucose levels falling below 40 mg/dL within the first 24 hours after birth, accompanied by symptoms such as seizures, lethargy, feeding refusal, respiratory distress, jitteriness, and hypotonia. Alternatively, it was also defined as blood glucose levels dropping below 25 mg/dL within the first four hours of life or below 35 mg/dL between four and 24 hours of life, without any symptoms [10]. A preterm baby was defined as an infant born before 37 weeks of pregnancy.

Data collection involved obtaining parental consent, recording baseline demographic information (age in hours, birth weight, gender, and gestational age), and random glucose levels. Blood glucose levels were checked using a digital glucometer (Freestyle, Abbott Laboratories, Chicago, IL) by the heel prick method under strict aseptic conditions, 30 minutes after the first feed and then at four-hour intervals until 24 hours. Hypoglycemic newborns underwent more frequent blood glucose measurements and received increased frequency of feeding or intravenous glucose according to standard protocols [10].

Data was recorded on a specially designed pro forma. Analysis, performed using SPSS for Windows, version 24.0 (IBM SPSS Statistics, Armonk, NY), computed frequencies and percentages for qualitative variables (gender and hypoglycemic/non-hypoglycemic babies) and presented mean±SD for quantitative variables (age, weight, gestational duration, and glucose levels). Stratification by age, weight, and gestational duration assessed their impact on hypoglycemic frequency, followed by post-stratification using the chi-squared test. Statistical significance was set at p≤0.05.

## Results

The study comprised 186 preterm neonates, with 47.3% (n=88) aged under four hours and 52.7% (n=98) aged four to 24 hours. Among them, 60.8% (n=113) were males, and 39.2% (n=73) were females. The mean gestational age at birth was 31.3±2.7 weeks, with an average birth weight of 2.1±0.3 kg. Hypoglycemia occurred in 31.7% (n=59) of neonates, with 52.5% (n=32) of them showing symptoms. Although hypoglycemia was slightly more prevalent in male infants than females (32.7%, 37/113, versus 30.1%, 22/73), the difference was not statistically significant (Table 1). The average age of hypoglycemia onset was 4.07±1.35 hours.

**Table 1:** Gender-wise stratification of hypoglycemia The chi-squared test was used with p≤0.05 being significant

Gender	Hypoglycemia		P-value
	Yes	No	
Male	37 (32.7%)	76 (66.3%)	0.709
Female	22 (30.1%)	51 (69.9%)	
Total	59 (31.7%)	127 (68.3%)	

There was a significant difference in the occurrence of hypoglycemia between the first four hours of life and the subsequent 20 hours (p=0.042) (Table 2). Symptomatic hypoglycemia was slightly less common in the 4-24-hour age group (48%, n=12) compared to the 0-4-hour age group (55.9%, n=19), but this variation was not statistically significant (p=0.549) (Table 3).

**Table 2:** Age-wise stratification of hypoglycemia The chi-squared test was used with p≤0.05 being significant

Age groups	Hypoglycemia		P-value
	Yes	No	
≤4 hours	35 (39.8%)	53 (60.2%)	0.042
4-24 hours	24 (24.5%)	74 (75.5%)	
Total	59 (31.7%)	127 (68.3%)	

**Table 3:** Age-wise stratification of symptomatic hypoglycemia The chi-squared test was used with p≤0.05 being significant

Age groups	Symptomatic hypoglycemia		P-value
	Yes	No	
≤4 hours	19 (55.9%)	15 (44.1%)	0.549
4-24 hours	12 (48%)	13 (52%)	
Total	31 (52.5%)	28 (47.5%)	

A statistically significant difference was observed in the frequency of hypoglycemia between infants born with gestational ages below 32 weeks and those born at or beyond 32 weeks. Infants born before 32 weeks of gestation were more susceptible to hypoglycemia (Table 4).

**Table 4:** Gestational-age-wise stratification of hypoglycemia The chi-squared test was used with p≤0.05 being significant

Gestational age	Hypoglycemia		P-value
	Yes	No	
≤32 weeks	33 (40.7%)	48 (59.3%)	0.02
>32 weeks	26 (24.8%)	79 (75.2%)	
Total	59 (31.7%)	127 (68.3%)	

Neonates weighing less than 2 kg exhibited a higher susceptibility to hypoglycemia (35.6%, 29/81, versus 28.6%, 30/105), although this disparity was not statistically significant (Table 5).

**Table 5:** Birth weight stratification of hypoglycemia The chi-squared test was used with p≤0.05 being significant

Birth weight	Hypoglycemia		P-value
	Yes	No	
>2.0 kg	30 (28.6%)	75 (71.4%)	0.293
≤2.0 kg	29 (35.8%)	52 (64.2%)	
Total	59 (31.7%)	127 (68.3%)	

Feeding refusal was the most common symptom of symptomatic hypoglycemia, affecting 80.6% (n=25) of newborns. Hypotonia was the least prevalent symptom, observed in 29% (n=9) of cases (Table 6).

**Table 6:** Signs and symptoms of symptomatic hypoglycemia in premature newborns (n=31)

Signs/symptoms	Number (%)
Feeding refusal	25 (80.6%)
Lethargy	11 (35.4%)
Seizures	17 (54.8%)
Jitteriness	22 (70.9%)
Respiratory distress	13 (41.9%)
Hypotonia	9 (29%)

## Discussion

Hypoglycemia, derived from the Greek words hypo (below or under), glykys (sweet), and haima (blood), was coined by Harris in the late 19th century [11]. The American Academy of Pediatrics (AAP) defines neonatal hypoglycemia as a plasma glucose level below 47 mg/dL (2.6 mmol/L) [1]. The intervention criteria for hypoglycemia vary based on clinical context and infant characteristics. For example, symptomatic infants with glucose levels below 40 mg/dL require immediate treatment, while asymptomatic, at-risk term formula-fed infants may need increased feeding frequency. Intravenous glucose is administered if levels drop below 25 mg/dL from birth to four hours or below 35 mg/dL from four to 24 hours [10].

Preterm neonates are at an elevated risk of hypoglycemia and its complications due to reduced glycogen and fat reserves, an inability to generate glucose via gluconeogenesis, heightened metabolic demands from a larger brain size, and inadequate counterregulatory responses to hypoglycemia [2]. Low-birth-weight infants, including both preterm and small-for-date babies, are especially prone to hypoglycemia, particularly within the first 24 hours after birth [12]. Given the lack of studies addressing both preterm birth and the first 24 hours of life in a single study, we aimed to investigate the prevalence of hypoglycemia in this specific population.

Hypoglycemia in neonates results from reduced glucose production or increased glucose utilization. This can occur due to insufficient glycogen and fat stores at birth, limiting gluconeogenesis, or excessive glucose use, such as in infants of mothers with insulin-dependent diabetes [13,14]. Extremely-low-birth-weight preterm infants face challenges because key enzymes for gluconeogenesis are expressed at low levels. Conditions such as intrauterine growth restriction (IUGR) and congenital disorders can further predispose infants to hypoglycemia [15]. Neonates have limited counterregulatory mechanisms against hypoglycemia, responding by reducing insulin secretion and increasing glucagon, epinephrine, growth hormone, and cortisol to mobilize glucose and fatty acids. However, persistent hypoglycemia can occur due to insulin overuse, poor gluconeogenesis, or failure of these counterregulatory mechanisms [2]. In this study, 31.7% of infants developed hypoglycemia within the first 24 hours of life. It was evident that very premature babies (gestational age of <32 weeks) were more susceptible to hypoglycemia. Additionally, it is noteworthy that these babies were more susceptible to hypoglycemia in the first four hours of life. Schaefer-Graf et al., in their study on the rate of hypoglycemia in large-for-gestational-age babies, found that 16% of infants developed hypoglycemia within the first 24 hours of life, with 9.2% occurring within the first hour itself [16].

Maternal diabetes, when well-controlled, may be protective against neonatal hypoglycemia. However, macrosomia remains an independent risk factor. Other consistent independent risk factors include cesarean section, lower gestational age, treatment for chorioamnionitis, and small for gestational age (SGA). More research is needed to confirm these findings and develop preventive measures [17]. For healthy term newborns, routine glucose screening lacks evidence-based support and can even interfere with successful breastfeeding. Healthcare professionals should prioritize avoiding unnecessary screening, proper glucose monitoring education, and promoting exclusive breastfeeding in this population [18].

In our study, males were more affected than females (32.7% versus 30.1%). Similar findings were observed in studies by Singh et al. (16.99% versus 13.33%) [19] and Bell et al. (100 males and 69 females) [20].

Hypoglycemia in newborns can present various symptoms. In our study, refusal to feed was the most prevalent symptom, affecting over 80% of symptomatic infants. This aligns with findings from previous studies by Dashti et al. [21] and Ibrahim et al. [9], which also identified feeding difficulties as the primary symptom. The consistent results across local and international studies suggest that feeding refusal may be a universal indicator of hypoglycemia in newborns.

This study has limitations. Firstly, it was conducted at a single medical center, potentially limiting the generalizability of findings to other populations or healthcare settings. Secondly, non-probability convenience sampling was used, which might not have captured a perfectly representative sample of the hospital's population. More studies with broader generalizability are needed to better understand the frequency and severity of hypoglycemia in preterm neonates, particularly within the first 24 hours of life.

In summary, hypoglycemia poses significant challenges and requires careful management. The definition and intervention criteria for hypoglycemia vary based on clinical context, with close monitoring and timely treatment being essential, especially in preterm infants. This study highlights the prevalence of hypoglycemia within the first 24 hours of life, particularly among premature babies, emphasizing the need for early detection and intervention. The observation of feeding refusal as a common symptom underscores its importance as a potential indicator of hypoglycemia in newborns. Further studies are warranted to deepen our understanding and improve management strategies for this critical condition in neonatal care.

## Conclusions

In our study population, about one-third of preterm infants experienced hypoglycemia within the first 24 hours. While the study hints at potential associations between earlier gestational age, lower birth weight, and male gender with a heightened risk of hypoglycemia, larger studies are required to confirm these trends. Close monitoring of blood glucose levels in all preterm infants, especially during the initial four hours, is essential to prevent complications. Further research with larger, more diverse cohorts and rigorous methodologies is necessary to validate these findings and enhance the understanding of hypoglycemia in preterm neonates, particularly within the initial 24 hours of life.
